# Changes in Hedonic Hunger and Problematic Eating Behaviors Following Bariatric Surgery: A Comparative Study of Roux-en-Y Gastric Bypass Versus Sleeve Gastrectomy

**DOI:** 10.3390/healthcare14010127

**Published:** 2026-01-04

**Authors:** Can Selim Yilmaz, Perim Fatma Turker

**Affiliations:** 1 Department of Nutrition and Dietetic, Faculty of Health Sciences, Baskent University, Ankara 06790, Turkey; csyilmaz@baskent.edu.tr; 2 Department of Nutrition and Dietetics, Faculty of Health Sciences, Acibadem Mehmet Ali Aydinlar University, Istanbul 34752, Turkey

**Keywords:** hedonic hunger, problematic eating behaviors, sleeve gastrectomy, Roux-en-Y gastric bypass, bariatric surgery

## Abstract

**Background/Objective**: This study aims to examine changes in hedonic hunger and problematic eating behaviors over a 24-week follow-up period after sleeve gastrectomy and Roux-en-Y gastric bypass and to compare differences between the two surgical procedures. **Methods**: This prospective observational study included 144 adults who underwent sleeve gastrectomy (*n* = 74) or Roux-en-Y gastric bypass (*n* = 70). Hedonic hunger was assessed using the Power of Food Scale, and problematic eating behaviors were evaluated with the Eating Disorder Examination Questionnaire. Data was collected one week before surgery and 24 weeks postoperatively through face-to-face interviews. **Results**: At the 24-week follow-up, participants who underwent gastric bypass had higher total Power of Food Scale scores than those who underwent sleeve gastrectomy (2.42 vs. 2.15), although reductions from baseline were not significantly different (−1.31 vs. −1.16, *p* = 0.136). Both procedures resulted in significant decreases in total Eating Disorder Examination Questionnaire scores (sleeve gastrectomy: 2.37 to 1.00, *p* < 0.01; gastric bypass: 2.41 to 1.36, *p* < 0.01), as well as in Eating Concern, Shape Concern, and Weight Concern subgroups. Reductions in Eating Disorder Examination Questionnaire total score and in Shape Concern and Weight Concern subgroups score were greater after sleeve gastrectomy (−1.37 vs. −1.05, *p* = 0.030). Total weight loss percentage was positively correlated with decreases in Eating Disorder Examination Questionnaire scores in both groups (*p* = 0.010) and was significantly associated with Power of Food Scale reductions only in sleeve gastrectomy (r = 0.163, *p* = 0.014). **Conclusions**: Both procedures reduce hedonic hunger and problematic eating behaviors, but the magnitude of change and its effect on weight loss may vary by surgical method.

## 1. Introduction

Sleeve gastrectomy (SG) and Roux-en-Y gastric bypass (RYGB) are the two most commonly performed bariatric procedures, each associated with substantial total weight loss, remission of obesity-related comorbidities and improvements in quality of life [1,2,3]. Despite these shared benefits, the procedures differ in their physiological, hormonal, and behavioral mechanisms, as well as in their anatomical alterations and potential postoperative risks. SG functions primarily as a restrictive procedure, reducing gastric volume and thereby limiting food intake and overall energy consumption. In contrast, RYGB incorporates both restrictive and malabsorptive components: a small gastric pouch restricts intake, while intestinal rerouting reduces nutrient absorption [4,5]. The surgeries also elicit distinct hormonal responses. SG is associated with reductions in ghrelin, contributing to decreased appetite and enhanced satiety. RYGB, through postoperative changes in glucagon-like peptide-1 (GLP-1), peptide-YY (PYY) and ghrelin, promotes satiety and improves insulin sensitivity [4,6]. Postoperative risks likewise vary between procedures. RYGB is more commonly associated with complications such as dumping syndrome, internal hernias and anastomotic leaks, whereas SG is more frequently linked to gastroesophageal reflux disease, staple-line leaks and strictures [7].

The choice between SG and RYGB should be individualized by considering not only comorbidities, body mass index (BMI), and surgical risk, but also the patient’s capacity to maintain long-term dietary and nutritional adherence. For instance, findings from a decision-tree analysis in patients with type-2 diabetes mellitus indicate that individuals with a diabetes duration longer than 5 years achieve higher remission rates with RYGB compared with SG. In contrast, for patients with shorter diabetes duration and a BMI ≥ 35.5 kg/m^2^, SG may be a more cost-effective option. Likewise, registry-based data consistently show that RYGB yields greater excess weight loss and higher rates of remission for comorbidities such as type-2 diabetes mellitus, hypertension, obstructive sleep apnea and gastroesophageal reflux over several years [8,9,10]. Conversely, SG is associated with lower rates of reoperation, readmission, and nutritional complications in observational studies, as well as lower long-term all-cause mortality in some meta-analyses. These factors may make SG preferable for patients with higher surgical risk or those who favor a less complex procedure [11,12,13]. Beyond comorbidity profiles and surgical risk, patients’ nutritional habits and their anticipated ability to adhere to postoperative dietary recommendations may also guide the choice between SG and RYGB. Evidence indicates that both procedures are generally associated with reductions in food cravings, emotional or external eating, and other maladaptive eating behaviors [14,15,16]. Notably, a recent meta-analysis of 42 studies reported no consistent association between postoperative daily energy intake and the degree of weight loss, suggesting that long-term outcomes may depend more on sustained behavioral and dietary adjustments, as well as nutritional quality, rather than early postoperative caloric intake alone [17]. Furthermore, it has been reported that patients with maladaptive eating patterns prior to surgery (e.g., binge eating, night eating or strong carbohydrate cravings) do not consistently show worse weight loss results post-surgery compared to those without such behaviors [18]. This suggests that preoperative eating patterns should not be used in isolation to determine the choice of surgical procedure. Instead, the patient’s motivation and capacity to maintain long-term behavioral changes, including portion control, balanced dietary intake, adequate protein consumption, and adherence to micronutrient supplementation, may be more critical for postoperative success.

Studies have reported that both SG and RYGB lead to reductions in hedonic hunger (the urge to eat for pleasure in the absence of physiological hunger) [19,20,21,22,23], as well as improvements in disordered eating behaviors [24,25]. Several factors may account for this reduction, including diminished food tolerance [26,27,28,29], gastrointestinal symptoms such as constipation, reflux, diarrhea, abdominal pain, indigestion and dumping syndrome [30,31], a shift toward healthier food choices [32,33,34], specific food preferences or avoidance behaviors [22], increased postprandial satiety, reduced hunger and alterations in taste perception [35]. Additionally, decreased food cue reactivity after surgery has been linked to reduced reward responses to energy-dense foods [19].

Beyond their medical and physical benefits, bariatric procedures also exert important psychosocial effects, including reductions in maladaptive eating behaviors, improvements in eating disorder (ED) symptoms, and decreases in anxiety and depressive symptoms [36,37]. Problematic eating behaviors (PEBs) are prevalent among individuals seeking bariatric surgery and may reach clinically significant ED severity in a subset of patients [38,39,40]. Although PEBs and ED symptoms generally decline following surgery, a substantial proportion of patients continue to experience these difficulties postoperatively, with more than 47% reporting PEBs and over 10% meeting criteria for an ED in some studies [38,41,42]. Eating behavior is a central determinant of both the development of obesity and postoperative percent total weight loss (%TWL). Postoperative changes in eating patterns are influenced by alterations in gastrointestinal physiology [19], and the persistence of PEBs or ED symptoms has been consistently linked to reduced %TWL following surgery [39,41,42,43].

Both SG and RYGB are broadly associated with reductions in hedonic hunger and improvements in problematic eating behaviors; however, the pattern and magnitude of these changes may differ between procedures. These variations are thought to arise from surgery-specific physiological mechanisms—such as alterations in gut–brain signaling and reward processing—as well as differential hormonal responses involving GLP-1, PYY, and ghrelin. Psychological factors, including the presence or remission of eating disorder symptoms, and behavioral adaptations such as avoidance of certain foods or diminished interest in eating, may further shape postoperative outcomes. Additionally, procedure-related differences in gastrointestinal symptoms (e.g., dumping syndrome), food tolerance, reinforcement processes, and eating patterns likely contribute to the distinct trajectories observed following SG versus RYGB [19,24,31,44,45,46,47,48,49,50].

Existing studies in the literature have typically examined either a single bariatric procedure or multiple procedures collectively, without conducting separate comparative analyses. Although research has evaluated hedonic hunger and problematic eating behaviors in patients undergoing SG or RYGB, relatively few studies have explored the bidirectional relationship between these constructs across different surgical modalities. Therefore, this study may contribute to the literature in terms of revealing the difference between the two bariatric surgery procedures and help to address the existing gap in the literature. This study aims to investigate changes in hedonic hunger and problematic eating behaviors over a 24-week follow-up period following SG and RYGB, and to evaluate potential differences between the two regimens.

## 2. Materials and Methods

### 2.1. Study Design, Participants and Procedures

This study was designed as a prospective observational study. The study was conducted with 152 adults (78 SG, 74 RYGB) who applied to bariatric center for weight management and metabolic surgery at two hospitals in Ankara, Turkey between March 2024 and February 2025. The inclusion criteria required participants to be at least 18 years of age, to have a BMI ≥ 35.0 kg/m^2^ with obesity-related comorbidities or a BMI ≥ 40.0 kg/m^2^, and to be scheduled to undergo SG or RYGB as a bariatric surgical intervention.

All procedures performed in studies involving human participants were in accordance with the ethical standards of the institutional and/or national research committee and with the 1964 Helsinki declaration and its later amendments or comparable ethical standards. This study was approved by Baskent University ethics committee (Project no: KA24/66; approval date: 28 February 2024). Informed written consent was obtained from each subject.

The required sample size was determined using G*Power version 3.1.9.7. Drawing on percentage estimates from previous studies, an effect size of 0.6, a power of 80%, and a significance level of 0.05 were applied, indicating a minimum sample size of 45 participants. To account for potential attrition, 78 individuals were recruited for the SG group and 74 for the RYGB group.

Prior to bariatric surgery, all patients were assessed by a multidisciplinary team, including a bariatric surgeon, bariatric physician, bariatric specialist nurse, dietitian and psychologist, and their medical, surgical, nutritional, physical and psychiatric history were assessed. Eight participants were excluded from the study for the following criteria: The presence of chronic conditions that could influence metabolic rate or appetite and/or the use of medications affecting these processes (two participants with uncontrolled hypothyroidism and two participants using antidepressant or antipsychotic medications); concurrent enrollment in another behavioral or obesity treatment program (one participant); engagement in above-average levels of physical activity (one participant); and loss to follow-up (two participants). After these exclusions, the final sample included 144 adults (74 SG, 70 RYGB) with comparable baseline characteristics.

SG and RYGB procedures were conducted using standard laparoscopic surgical techniques. Following surgery, all patients received routine instructions to adhere to a staged dietary progression, beginning with a liquid diet during the first two weeks, transitioning to a soft diet for the subsequent two weeks, and ultimately advancing to a solid diet [51].

### 2.2. Data Collection Process

**Figure 1:**
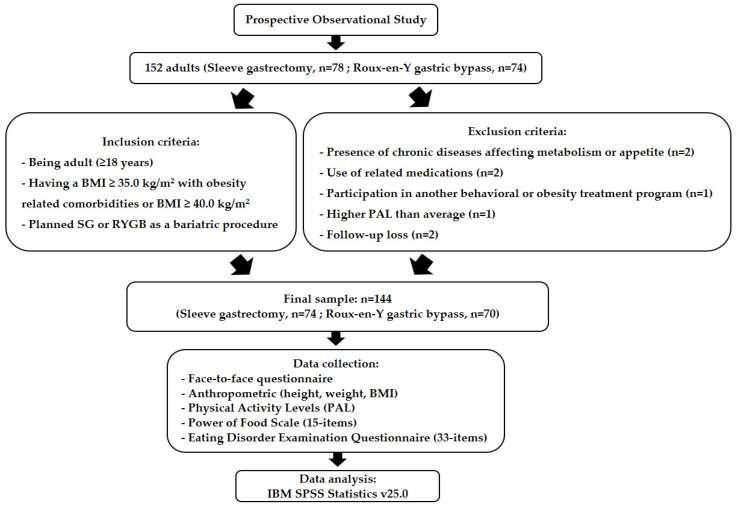
Flowchart of the study.

Research data were obtained one week prior to surgery (T0) and again at the 24-week postoperative follow-up (T1) through face-to-face interviews. Participants completed a questionnaire that included a sociodemographic and clinical information form, anthropometric measurements (body weight and height), assessment of physical activity level (PAL), the Power of Food Scale (PFS) and the Eating Disorder Examination Questionnaire (EDE-Q). The flowchart of the study is presented in Figure 1.

### 2.3. Measures

#### 2.3.1. Demographic, Anthropometric, and Physical Activity Assessments

One week before surgery, participants completed a structured questionnaire including multiple-choice items on socio-demographic characteristics, administered by the research team through face-to-face interviews. Anthropometric data were collected and documented by researchers. Height (cm) was assessed using a calibrated stadiometer (Seca, Hamburg, Germany), with participants barefoot and dressed in light clothing (shorts and a T-shirt), while body weight (kg) was measured with a calibrated digital scale (Simbo SBS-4439, Istanbul, Turkey). Anthropometric measurements were obtained in the morning after an overnight fast of at least eight hours at both data collection time points. BMI was subsequently calculated using the standard formula (kg/m^2^). Participants’ PAL was evaluated using a 24 h physical activity assessment form [52].

#### 2.3.2. Hedonic Hunger

The PFS, a 15-item instrument developed by Lowe and Butryn [53], was administered one week before surgery and again at 24 weeks postoperatively. The validated Turkish adaptation of the scale was used in this study [54]. The PFS evaluates individuals’ responsiveness to food across three domains: Food Available (FA), referring to food that is accessible but not physically present; Food Present (FP), indicating food that is present but not yet tasted; and Food Tasted (FT), representing food that has been tasted but not consumed. Items are rated on a 5-point Likert scale (1 = strongly disagree to 5 = strongly agree), and total scores are calculated as the mean of the three subgroup scores [21,53]. Higher PFS scores indicate greater levels of hedonic hunger.

#### 2.3.3. Problematic Eating Behaviors

The 33-item EDE-Q, developed by Fairburn & Beglin in 1994 [55], is used to measure basic eating disorder behaviors and cognitive symptoms a week before and 24 weeks after surgery. The present study used the validated Turkish version of the EDE-Q [56]. The questions in the scale are rated using a 7-point Likert scale ranging from 0 to 6. The EDE-Q includes 4 subgroups: Dietary restraint (R), eating concerns (EC), weight concerns (WC) and shape concerns (SC). Total EDE-Q score is determined by averaging the four subgroup scores. The EDE-Q is a widely utilized instrument for assessing eating-related psychopathology in individuals undergoing bariatric surgery and has demonstrated robust validity and reliability in this population [57]. Higher EDE-Q scores indicate greater severity of problematic eating behaviors. The cut-off value for the total EDE-Q score in normal weight populations has been reported to be 2.5–2.8 on average, depending on different samples [58,59]. In this study, a mean score threshold of ≥2.5 was used to identify the presence of PEBs.

### 2.4. Statistical Analysis

The study data were analyzed using IBM SPSS Statistics version 25.0. Descriptive statistics (mean, standard deviation, frequency, and percentage) were calculated. Data normality was assessed using the Shapiro–Wilk and Kolmogorov–Smirnov tests. For normally distributed variables, group comparisons were conducted with one-way ANOVA, followed by Bonferroni-adjusted post hoc tests when applicable. Associations between categorical variables were examined using the chi-square test, and relationships between continuous variables were assessed using Pearson’s correlation coefficient. Regression analyses were conducted to evaluate the independent effects of genders on outcomes of interest. A *p*-value < 0.05 was considered statistically significant.

## 3. Results

The socio-demographic characteristics of the participants are shown in Table 1. The study included 144 patients who underwent SG (*n* = 74; 55% female; mean age 35.65 ± 15.42 years) or RYGB (*n* = 70; 70% female; mean age 32.40 ± 7.95 years). Although SG and RYGB patients were broadly comparable in terms of age, BMI, comorbidities, physical activity, and preoperative weight, the RYGB group included a significantly higher proportion of female participants. A regression analysis regarding the role of gender was also conducted, and no significant effect was observed. These similarities in baseline status support the comparability of postoperative changes between the two procedures.

**Figure 2:**
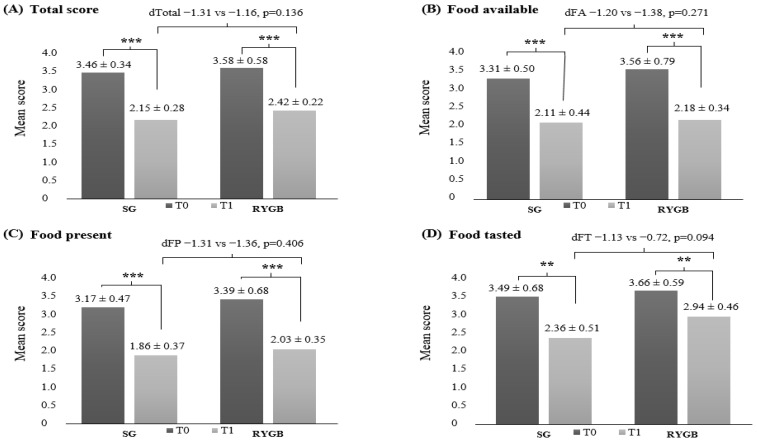
Total and subgroup mean scores of PFS according to bariatric regimens. Data presented as mean ± SD; Asterics denote significant differences over time (repeated measures ANOVA with post hoc Bonferroni, *** *p* < 0.001, ** *p* < 0.01).

Total and subgroup PFS scores by surgical procedure are presented in Figure 2. At baseline (T0), no significant differences were observed between SG and RYGB in overall PFS scores or in any of the subgroups. By the 24-week follow-up (T1), both procedures showed significant reductions in total PFS and all subgroup scores compared with baseline. Although mean total PFS, FP, and FT scores were slightly higher in the RYGB group than in the SG group at T1, these differences were not statistically significant. Similarly, the magnitude of change in PFS scores from T0 to T1 did not differ significantly between the two procedures. Clinically, the parallel reduction in hedonic hunger across procedures suggests that both SG and RYGB effectively diminish reward-driven eating during the early postoperative period. However, the relatively higher hedonic hunger observed in the RYGB group (despite being no significant) may indicate a need for earlier behavioral reinforcement, tailored nutritional counseling, or monitoring of food-cue reactivity in this procedure.

**Figure 3:**
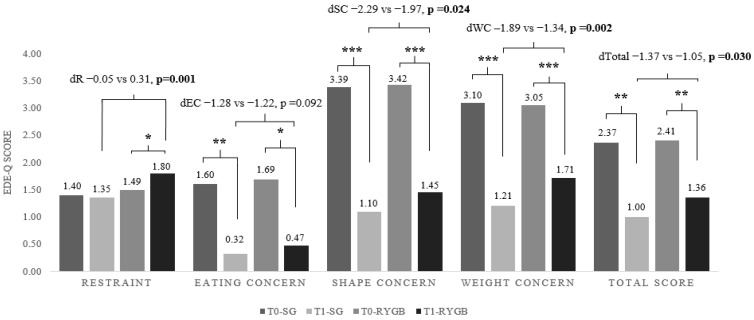
Total and subgroup mean scores of EDE-Q according to bariatric regimens. Data presented as mean ± SD; Asterics denote significant differences over time (paired *t* test and Student *t* test, (* *p* < 0.05, ** *p* < 0.01, *** *p* < 0.001).

Total and subgroup mean EDE-Q scores by bariatric regimen are shown in Figure 3. Both groups demonstrated a significant reduction in total EDE-Q scores from T0 to T1 (SG: 2.37 ± 0.50 to 1.00 ± 0.24, *p* < 0.01; RYGB: 2.41 ± 0.69 to 1.36 ± 0.32, *p* < 0.01). All subgroup scores declined significantly in both groups, except for the Restraint subgroup in the RYGB group. Unlike other domains, restraint scores increased significantly following RYGB (1.49 ± 1.20 to 1.80 ± 0.69, *p* = 0.027). When comparing changes between procedures, the reductions in total EDE-Q scores and in the Restraint, Shape Concern, and Weight Concern subgroups were significantly greater in the SG group than in the RYGB group. The greater postoperative decline in problematic eating behaviors in SG patients suggests that SG may promote a more favorable behavioral adjustment in the short term. In contrast, increased restraint among RYGB patients may reflect heightened vigilance toward eating or reliance on restrictive strategies to meet postoperative demands. Clinicians may therefore need to provide targeted behavioral support to RYGB patients to prevent rigid, unsustainable restraint patterns that could predispose to later problematic eating or weight regain.

The prevalence of PEB, defined as an EDE-Q total score ≥ 2.5, is presented in Table 2. At baseline, PEB prevalence was comparable between regimens (SG: 56.8%; RYGB: 64.3%). By the 24-week follow-up (T1), these rates declined markedly in both groups, reaching 5.4% in SG and 17.1% in RYGB patients. Although no difference was observed at T0, the prevalence of PEB at T1 was significantly higher in the RYGB group (*p* = 0.012). Similarly, baseline subgroup-specific PEB prevalence did not differ between regimens. However, at T1, participants who underwent RYGB exhibited significantly higher PEB prevalence in the Eating Concern, Shape Concern, and Weight Concern subgroups compared with those who underwent SG (*p* = 0.040, *p* = 0.021, and *p* = 0.010, respectively), while no difference was observed for the Restraint subgroup. These findings suggest that SG may facilitate a more pronounced reduction in disordered eating risk during the early postoperative period, whereas RYGB patients may require more intensive behavioral follow-up to address persistent concerns related to eating, shape, and weight. Incorporating structured psycho-nutritional support for RYGB patients may help mitigate the persistence of these cognitive and emotional eating disturbances.

Table 3 shows procedure-specific associations between %TWL and changes in PFS and EDE-Q scores. In SG patients, reductions in total PFS, FA, and FP scores were positively associated with %TWL, suggesting that improvements in hedonic hunger are closely linked to weight loss. In RYGB patients, significant associations were limited to FA and FP decreases, with no significant relationship between total PFS improvement and %TWL. For EDE-Q, SC and WC subgroup score reductions were related to %TWL only in SG patients. These findings imply that SG patients may experience a more behaviorally mediated weight-loss pathway, with reductions in hedonic hunger and cognitive eating disturbances functioning as key contributors. In contrast, weight loss after RYGB may rely more heavily on anatomical and physiological mechanisms, with weaker behavioral correlations. This supports the use of targeted behavioral interventions after SG to maximize weight-loss outcomes, while emphasizing continued structured follow-up for RYGB patients to align behavioral change with surgical efficacy.

Regression analysis on the role of gender in hedonic hunger and eating psychopathology is shown in Table 4. Overall, female gender was associated with a modest effect on both hedonic hunger and eating behavior; however, these associations did not reach statistical significance in any of the models. This suggests that, within the context of the current analyses, gender did not independently contribute meaningfully to variations in these outcomes.

## 4. Discussion

The present study evaluated changes in hedonic hunger among patients undergoing SG and RYGB. At baseline (T0), total and subgroup PFS scores did not differ significantly between the two surgical groups. By the 24-week follow-up (T1), both procedures were associated with significant reductions in total PFS and all subgroup scores. Although patients who underwent RYGB exhibited slightly higher total, FP, and FT scores at T1 compared with SG, these differences were not statistically significant. Likewise, the magnitude of reduction in total and subgroup PFS scores from T0 to T1 was similar between the procedures. Early postoperative changes in hedonic hunger appear to follow a consistent pattern across bariatric procedures. Several studies have demonstrated marked reductions in hedonic hunger similar to those observed in our study during the first 6–12 months after surgery, a period characterized by rapid weight loss, altered gut hormone secretion, and reduced physiological drive to eat. Contemporary evidence suggests that this early decline is robust across both SG and RYGB, with substantial reductions reported in PFS scores within the first postoperative year [19,20,21,22,33,60]. These improvements are attributed to increases in satiety hormones and decreases in orexigenic signals, which collectively diminish the rewarding value of food. However, long-term patterns of hedonic hunger after bariatric surgery appear more heterogeneous than the pronounced early postoperative improvements. Although substantial decreases are consistently observed during the first year, several long-term studies suggest that this improvement may attenuate or partially reverse over time. In a 24-month follow-up of adolescents undergoing RYGB, Cushing et al. [60] demonstrated that hedonic hunger, declined markedly during the first 18 months but increased again at 24 months in parallel with modest BMI regain. Similar trends have been observed in adults. In a 13-year follow-up, Nymo et al. [49] found that individuals with suboptimal long-term weight outcomes exhibited higher hedonic hunger and stronger reward responses to palatable foods compared with those maintaining successful weight loss. Collectively, these findings indicate that although hedonic hunger declines substantially after surgery, a subset of patients may experience a late postoperative resurgence, particularly those vulnerable to weight regain. This underscores the importance of sustained behavioral and nutritional support to prevent the re-emergence of reward-driven eating beyond the first postoperative year.

The higher PFS, FP, and FT scores observed after RYGB in this study may be explained by its more pronounced effects on gut–brain signaling and food reward pathways. RYGB elicits greater increases in GLP-1 and PYY compared with SG, which can heighten sensitivity to food cues despite enhancing satiety [61,62]. Neuroimaging studies also suggest that RYGB produces stronger alterations in mesolimbic reward responses to palatable foods than SG [45,63]. These combined hormonal and neurobehavioral changes may contribute to elevated hedonic hunger and cue reactivity reflected in higher PFS scores following RYGB. RYGB produces marked alterations in gut hormone secretion—including GLP-1, PYY, and ghrelin—and modifies nutrient transit, collectively influencing satiety and reward-related eating. Although some studies report an initial reduction in hedonic hunger following RYGB, this effect may plateau by 24 weeks or become attenuated by psychological factors such as increased awareness of dietary restrictions and the challenges of adapting to new eating behaviors. In contrast, SG typically leads to a more gradual decline in ghrelin levels and induces fewer anatomical and physiological changes in the gastrointestinal system, which may contribute to comparatively smaller reductions in hedonic hunger during the early postoperative period [19,47]. Differences in postoperative dietary requirements may further account for the observed patterns. The stricter dietary limitations and greater malabsorption associated with RYGB may increase psychological preoccupation with food, potentially contributing to higher PFS scores. Conversely, SG generally involves fewer restrictions related to food texture and portion size, which may reduce eating-related stress and mitigate hedonic cravings [19,44,45,46].

Taste alteration after bariatric surgery is a well-documented phenomenon and is considered an important mechanism influencing postoperative eating behavior [64]. Both RYGB and SG have been shown to modify taste perception through changes in gut–brain signaling, altered expression of taste receptors, and shifts in reward processing [65]. Postoperative increases in GLP-1, PYY, and changes in bile acid metabolism are thought to modulate central gustatory pathways, leading to reduced preference for high-fat and high-sugar foods and increased sensitivity to basic tastes such as sweetness and bitterness [62]. These alterations may contribute to early reductions in hedonic drive and improved dietary quality; however, they may also lead some patients to experience heightened aversions, reduced enjoyment of certain foods, or compensatory shifts toward more palatable textures [66]. A recent cross-sectional study [67] including patients 1 year after RYGB, SG, or One-anastomosis gastric bypass (OAGB) reported that most participants experienced changes in taste perception, cravings, and enjoyment of flavors, with RYGB and OAGB being associated with a more pronounced reduction in craving for sweet and fatty foods compared with SG. Similarly, in a controlled study [68], patients undergoing bariatric surgery commonly self-reported decreased preference for energy-dense foods and a shift toward healthier eating, which was linked more to altered hedonic responses than purely changed taste sensitivity. Neuroimaging and behavioral studies further suggest that changes in taste-reward processing (particularly reduced striatal activation in response to highly palatable stimuli) may partially mediate postoperative reductions in maladaptive eating patterns [45,69,70]. Overall, postoperative taste alterations may represent a complex but clinically meaningful factor that shapes food preferences, eating behavior, and long-term adherence to dietary recommendations.

The present study examined changes in PEBs among patients undergoing SG and RYGB, as assessed by the EDE-Q. Both procedures were associated with significant reductions in total EDE-Q scores from T0 to T1. Similarly, all subgroup scores declined significantly in both groups, except for the Restraint subgroup in RYGB patients, for whom scores increased significantly at T1. These findings are consistent with previous studies reporting reductions in PEBs and broader eating disorder symptoms following both SG and RYGB [24,38,41,42,71,72,73,74]. Gero et al. [73], noted significant decreases in total and subgroup EDE-Q scores over 12 months in patients who underwent RYGB. Similarly, Çalışır et al. [74] reported substantial decreases in EDE-Q total and subgroup scores at both 6 and 12 months following SG. Nasirzadeh et al. [72] also observed significant declines in EDE-Q scores during the first postoperative year after both procedures. However, it has documented increases in EDE-Q scores beyond the first postoperative year, suggesting a potential resurgence of eating pathology over time. Researchers have noted that the EDE-Q captures general eating disorder symptoms rather than surgery-specific behaviors, which may contribute to these observed increases. Similarly, Morseth et al. [58] reported continuous reductions in EDE-Q total scores during the first 24 months following RYGB, followed by increases at longer-term follow-up.

In the present study, reductions in total EDE-Q scores and in the R, SC, and WC subgroups were significantly greater among SG patients, indicating a more pronounced early postoperative improvement in eating psychopathology compared with RYGB. These differences may reflect procedure-specific physiological and behavioral mechanisms. SG and RYGB produce distinct gastrointestinal symptom profile. Such symptom–behavior interactions likely shape postoperative psychological and behavioral adaptations. For example, RYGB patients may adopt more restrictive or hypervigilant eating patterns to avoid symptom onset, which may influence the trajectory of EDE-Q subgroup scores [24,31,48]. SG, by contrast, tends to produce fewer gastrointestinal adverse effects, which may enable more flexible eating patterns and facilitate greater early improvements in disordered eating behaviors. The relatively limited malabsorption and lower incidence of postprandial complications after SG may also support smoother behavioral adaptation and thus contribute to the more substantial reductions in eating psychopathology observed [49,50].

The increase in the Restraint subgroup score among RYGB patients in our study may reflect intentional dietary restriction driven by reduced gastric capacity and the risk of dumping syndrome when overeating. Bond et al. [48] similarly reported that high-risk eating behaviors (e.g., consuming food beyond satiety or eating high-sugar and high-fat foods) were more strongly associated with dumping syndrome and gastrointestinal distress after RYGB than after SG, a pattern that may reinforce more restrictive or vigilant eating behaviors in RYGB patients.

A key consideration in interpreting postoperative changes in restraint is the distinction between adaptive restraint, which reflects purposeful dietary control consistent with surgical goals, and maladaptive cognitive restraint. After bariatric surgery, increased restraint (particularly among RYGB patients) may in many cases represent adaptive postoperative behavioral regulation, such as intentional portion control, avoidance of high-sugar foods to prevent dumping, and adherence to nutritional recommendations. This form of restraint is generally associated with better weight-loss outcomes and improved dietary quality [38]. However, restraint can also represent maladaptive cognitive restraint, characterized by rigid control, fear of eating, and over-restriction, which has been linked to loss-of-control eating, grazing, and poorer psychological outcomes in bariatric populations [75,76]. Evidence suggests that patients who experience more frequent or intense gastrointestinal symptoms (more typical after RYGB) may be particularly vulnerable to developing maladaptive forms of restraint [77,78]. Thus, the increase in restraint observed in RYGB patients may reflect a combination of beneficial postoperative vigilance and potentially problematic over-control, underscoring the clinical importance of differentiating between these patterns.

This study also examined the relationship between postoperative %TWL and changes in PFS and EDE-Q scores. Among SG patients, greater weight loss was significantly associated with larger reductions in the PFS total score, while a similar but non-significant trend was observed in the RYGB group. Subgroup analyses indicated that decreases in FA and FP scores were significantly correlated with %TWL in both procedures, whereas reductions in SC and WC scores were significantly associated with weight loss only among SG patients. These results are consistent with previous research demonstrating positive correlations between %TWL and improvements in EDE-Q scores [73,75]. It has been suggested that postoperative weight loss may contribute to improvements in eating disorder symptomatology, and that nutritional counseling may further support these changes [79]. Supporting these findings, Ribeiro et al. [80] reported a significant positive correlation between reductions in the FT subscale of the PFS and %TWL among SG patients at 11–18 months of follow-up. Similarly, Makaronidis et al. [19] found that early postoperative decreases in the FA and FP subscales predicted greater weight loss at 12–24 months. These results highlight the potential role of hedonic-hunger–related domains as proximal determinants of long-term weight trajectories. Neurobehavioral evidence also indicates that both procedures reduce reward responses to energy-dense foods [80], consistent with the observed associations between FA/FP scores and %TWL.

In this study, the stronger correlation between reductions in PFS total score and %TWL in SG may be explained by procedure-specific hormonal effects. SG induces a sustained reduction in ghrelin levels, which may more directly link decreases in hedonic hunger with postoperative weight loss. In contrast, weight loss after RYGB is driven primarily by enhanced satiety and malabsorptive mechanisms, which can occur largely independent of changes in hedonic hunger [8,11].

In the present study, the association between weight loss and reductions in EDE-Q-SC and EDE-Q-WC scores appeared stronger in SG patients. This may reflect the more frequent occurrence of adverse gastrointestinal symptoms after RYGB, which can lead to rigid dietary restraint independent of changes in body image. In contrast, SG is associated with fewer severe gastrointestinal effects, potentially allowing improvements in body image and disordered-eating cognitions to align more closely with weight loss [81,82].

The strength of these relationships may vary depending on the bariatric procedure performed, underscoring the need for procedure-specific postoperative monitoring and support. Regular assessments at defined intervals before and after bariatric surgery, with consideration of differences between surgical procedures, utilizing validated instruments, may play a critical role in detecting emerging issues that could adversely affect weight maintenance following surgery. Additionally, cognitive-behavioral approaches, especially post-op, can improve eating psychopathology and can support weight outcomes. Existing evidence underscores the importance of distinguishing early postoperative improvements from later fluctuating trajectories. While hedonic hunger and problematic eating behaviors declines substantially during the rapid weight loss phase, partial re-surgences may occur in the longer term. This pattern highlights the need for ongoing behavioral support aimed at managing reward-driven eating and eating behaviors beyond the first year after surgery. Long-term individualized follow-up that includes dietary counseling and psychological support may be beneficial for maintaining weight loss following bariatric surgery. It may also help to clarify differences in outcomes between surgical procedures.

A notable strength of this study is its direct comparison of hedonic hunger and eating-related psychopathology across two distinct bariatric procedures, an approach that has been overlooked in prior research. By evaluating SG and RYGB separately rather than aggregating surgical modalities, the study may provide a clearer understanding of procedure-specific behavioral trajectories. The simultaneous assessment of hedonic hunger, problematic eating behaviors, and their associations with weight loss may offer a more comprehensive behavioral profile than studies focusing on a single construct. Additionally, the use of validated psychometric instruments and a standardized follow-up timeframe enhances the reliability of the findings and strengthens the contribution of this study to the existing literature.

This study has a number of limitations that may guide future research designs. Although the sample size is large enough to detect trends within the group of adult patients with bariatric surgery, it is not large enough to make firm inferences about the total population of each bariatric surgery procedure. Furthermore, the outcome data in this study are limited to 24-week follow-up. Longer-term prospective studies with larger sample sizes are needed to more accurately evaluate the sustained effects of these variables. While the PFS and EDE-Q are among the most widely used self-report instruments for assessing hedonic hunger and PEBs, direct observations or assessments involving actual food exposure could offer more precise measurements. Although the 24 h physical activity assessment form is suitable for determining physical activity level, their inherent susceptibility to recall and reporting bias is a potential limitation. Furthermore, heterogeneity in study design, sample characteristics, assessment tools, and follow-up durations across existing research complicates direct comparisons and synthesis of findings.

## 5. Conclusions

This study demonstrated that both SG and RYGB led to significant reductions in hedonic hunger and problematic eating behaviors within the first 24 postoperative weeks. Although the magnitude of improvement in hedonic hunger was comparable between procedures, SG yielded greater reductions in several domains of problematic eating behaviors, including Restraint, Shape Concern, Weight Concern, and the global EDE-Q score. The prevalence of problematic eating behaviors also declined markedly in both groups, with lower rates observed after SG at follow-up. Associations between improvements and percentage total weight loss were more consistent in SG patients, particularly for reductions in hedonic hunger and concerns related to shape and weight. Gender did not independently influence postoperative behavioral outcomes. Overall, these findings indicate that both procedures produce favorable short-term changes in hedonic hunger and eating behavior, with SG showing comparatively greater improvements in specific eating domains.

## Figures and Tables

**Table 1:** Characteristics of the participants.

	SG	RYGB	*p*
Age (years)	35.6 ± 15.4	32.4 ± 7.9	0.142
Females, %	55.6	70.0	0.041 *
Married, %	60.5	55.7	0.524
Presence of chronic disease, %	24.3	28.6	0.218
Insulin resistance, %	14.9	17.1	0.150
Cardiovascular diseases, %	10.8	15.7	0.056
Arthritis diseases, %	6.8	8.6	0.110
Physical activity level	1.48 ± 0.4	1.43 ± 0.2	0.250
Body weight at T0, kg	110.1 ± 12.5	106.3 ± 20.3	0.096
Body weight at T1, kg	85.2 ± 11.2	80.7 ± 14.1	0.082
Mean weight loss, %	22.6 ± 6.1	24.1 ± 5.4	0.125
BMI at T0, kg/m^2^	42.1 ± 4.0	41.4 ± 4.5	0.351
BMI at T1, kg/m^2^	32.0 ± 4.1	29.8 ± 4.2	0.145

Chi square test, * *p* < 0.05. Continuous variables are presented as mean ± SD.

**Table 2:** The prevalence of PEB according to EDE-Q cut-off score.

	Presence of PEBs ^¥^					
	T0, *n* (%)			T1, *n* (%)		
EDE-Q	SG	RYGB	*p*	SG	RYGB	*p*
Restraint	15 (20.3%)	18 (25.7%)	0.160	11 (14.9%)	13 (18.6%)	0.098
Eating concern	9 (12.2%)	13 (18.6%)	0.096	3 (4.1%)	8 (11.4%)	0.040 *
Shape concern	52 (70.3%)	54 (80.0%)	0.240	10 (13.5%)	16 (22.3%)	0.021 *
Weight concern	59 (79.7%)	60 (85.7%)	0.110	8 (10.8%)	15 (21.4%)	0.010 *
Total	42 (56.8%)	45 (64.3%)	0.168	4 (5.4%)	12 (17.1%)	0.012 *

Chi-square, * *p* < 0.05; ^¥^ ≥2.5 score of EDE-Q define presence of PEBs.

**Table 3:** Associations between percentage of total weight loss and reductions in PFS and EDE-Q scores at the 24-week follow-up.

	The Percentage of Total Weight Loss			
	SG		RYGB	
	r	*p*	r	*p*
PFS total	0.163	0.014 *	0.146	0.120
Food available	0.255	0.040 *	0.186	0.024 *
Food present	0.126	0.020 *	0.205	0.046 *
Food tasted	0.086	0.095	0.195	0.110
EDE-Q total	0.124	0.010 *	0.122	0.010 *
Restraint	0.025	0.424	0.179	0.138
Eating concern	0.140	0.254	0.088	0.470
Shape concern	0.167	0.042 *	0.057	0.637
Weight concern	0.094	0.037 *	0.022	0.854

Pearson r test, * *p* < 0.05.

**Table 4:** Regression analysis on the role of gender in hedonic hunger and eating psychopathology.

	Females			
	SG		RYGB	
	β	*p*	β	*p*
PFS Total	0.092	0.382	0.102	0.287
PFS—FA	0.045	0.552	0.053	0.472
PFS—FP	0.051	0.438	0.074	0.302
PFS—FT	0.060	0.368	0.086	0.297
EDE-Q Total	0.067	0.417	0.079	0.270
EDE-Q—R	0.056	0.452	0.063	0.365
EDE-Q—EC	0.044	0.540	0.061	0.374
EDE-Q—SC	0.075	0.431	0.097	0.276
EDE-Q—WC	0.069	0.465	0.082	0.290

SG: Sleeve gastrectomy; RYGB: Roux-en-Y gastric bypass.

## Data Availability

The data presented in this study are available on request from the corresponding author. The data are not publicly available due to privacy or ethical restrictions.

## Abbreviations

The following abbreviations are used in this manuscript:

SG	Sleeve gastrectomy
RYGB	Roux-en-Y gastric bypass
BMI	Body mass index
GLP-1	Glucagon-like peptide-1
PYY	peptide-YY
ED	Eating disorder
PEBs	Problematic eating behaviors
%TWL	Percent total weight loss
PAL	Physical activity Level
PFS	Power of Food Scale
EDE-Q	Eating Disorder Examination Questionnaire
T0	Baseline, one week before surgery
T1	24 weeks after surgery
BW	Body weight
